# The Impact of the COVID-19 Pandemic and Lockdowns on Sex Workers in West Bengal, India

**DOI:** 10.1007/s10900-025-01452-y

**Published:** 2025-03-10

**Authors:** Katherine A. Lewis, Protim Ray, Emma Janibekyan, Niharika Kaushik, Darshna Anigol, Denise Tieu, Zhenyi Luo, Leonel Hernandez, Asim Sen, Suchith Kumar, Anne E. Fehrenbacher, Dallas Swendeman

**Affiliations:** 1 Department of Population and Public Health Sciences, Keck School of Medicine, University of Southern California, 1845 N Soto St, Los Angeles, CA 90032 USA; 2 Durbar Mahila Samanwaya Committee, 12/5 Nilmoni Mitra Street, Kolkata, West Bengal India; 3 Department of Epidemiology, University of California, 10833 Le Conte Ave, Los Angeles, CA 90095 USA; 4 School of Public Health, University of California, 2121 Berkeley Way Berkeley, Berkeley, CA 94704 USA; 5 School of Medicine, University of California, 4610 X St Sacramento, Davis, CA 95817 USA; 6 SK Professional Translation and Transcription Services, Mysore, Karnataka India; 7 Semel Institute for Neuroscience and Human Behavior, Center for Community Health, University of California, 760 Westwood Plaza, Los Angeles, CA 90024 USA

**Keywords:** Sex Work, COVID-19, Qualitative, India, Stress Proliferation

## Abstract

India’s COVID-19 lockdowns were among the strictest globally, and sex workers were uniquely impacted. Extremely limited literature has examined pandemic consequences on sex workers. We use a qualitative approach to examine the impact of the COVID-19 pandemic and lockdowns on the lives and livelihoods of sex workers in urban West Bengal, India. Cisgender female and male, and transgender female sex workers (*N* = 40) participated in individual in-depth interviews. Interviews were coded using inductive thematic coding. Nine themes arose from the data: (1) COVID-19 pandemic and lockdowns, (2) Social isolation, (3) Lack of customers, (4) Financial stress, (5) Decreased negotiating power, (6) Food insecurity, (7) Receiving support, (8) Providing support, and (9) Mental health. We propose a Gendered Stress Proliferation Model incorporating Pearlin et al.’s 1997 conceptualization of stress proliferation and Swendeman, Fehrenbacher et al.’s 2018 gendered stress process model. In this model, primary stressors of COVID-19 pandemic and lockdowns proliferated into secondary stressors across multiple domains (social isolation, lack of customers, financial stress, food insecurity, reliance on support, decreased negotiating power). All of these pathways were shaped by pre-existing vulnerabilities and systems of intersectional marginalization. These stressors had significant mental health impacts including feelings of depression and anxiety. Food insecurity has implications for physical health outcomes, and financial stress coupled with decreased negotiating power has implications for sexual health, potentially placing sex workers at increased risk of sexually transmitted infections and HIV. Gendered Stress Proliferation may be a useful framework to examine how marginalized populations uniquely experience population-level crises.

**Supplementary Information:**

The online version contains supplementary material available at 10.1007/s10900-025-01452-y.

## Introduction

### Sex Work and COVID-19 in India

Although sex work is not criminalized in India, legal restrictions, socio-cultural stigma, and occupational risks persist [1–5]. These factors create and reinforce disparities both in health outcomes (e.g., HIV and mental health) and related social determinants of health such as financial stability, housing security, incarceration, violence, and educational attainment [2, 3, 5]. In response to these challenges, the Durbar Mahila Samanwaya Committee (Durbar) was founded as a community-based organization focused on an empowerment-based approach to promote the health and rights of sex workers in West Bengal in partnership with and then leading STI/HIV intervention programs [6–9]. Durbar’s empowerment-based approach has been recognized as a WHO Model STI/HIV prevention program, and a significant body of literature has documented Durbar’s success in increasing condom use, decreasing HIV rates, and promoting the health and rights of community members [6–8, 10, 11].

India’s first COVID-19 case was recorded on January 30th, 2020, and the country entered a strictly enforced national lockdown in March 2020, which proved to be one of the largest and most restrictive lockdowns globally [12–15]. This lockdown continued until May 2020, and during this time, stay at home isolation and social distancing were strictly enforced with no public transportation running and only essential travel outside the home permitted during certain hours [12–14]. After the first lockdown was lifted, a normalization period followed, but a devastating second wave of COVID-19’s Delta variant in the spring of 2021 inspired a second lockdown period from March to May 2021 [13, 16, 17]. This second lockdown was not as strict as the first and was enforced as state-to-state restrictions rather than a national level policy [13, 14].

At its peak, India confirmed over 2.7 million COVID-19 cases and 28,000 deaths in one week during the Delta wave [18]. Cumulatively, from January 2020 to Spring 2023, India experienced nearly 45 million confirmed cases and over 500,000 deaths, though the true death toll has been estimated to be 6–7 times higher than official reports [18–20]. In a recent study using national data representing a quarter of India’s population, Gupta et al. found that life expectancy at birth in India declined by 2.6 years from 2019 to 2020, and that females experienced a 1-year greater decline in life expectancy compared to males, in contrast with global trends. This study also found that life expectancy declines were highest among marginalized castes and religious groups, highlighting the relationship between social disadvantage and disproportionate COVID-19 impacts. When applied to India’s total population, this mortality trend suggests 1.19 million excess deaths in 2020 alone [21]. The COVID-19 pandemic and lockdowns exacerbated existing economic disparities [12], and sex workers were particularly impacted due to preexisting vulnerabilities and the in-person nature of their work [1, 2, 5, 13, 22].

Considering the severity of the COVID-19 pandemic and lockdowns as well as the unique risks faced by sex workers, the importance of understanding and addressing the needs of this population has been established [2, 5, 23, 24]. Globally, studies have documented significant financial distress, decreased access to support services, pivots towards online sex work, increased experiences of police violence, mental health concerns, and decreased access to HIV prevention services among sex worker populations [25–30]. However, due to the uniquely strict nature of the COVID-19 lockdowns in India [15], findings from other countries are likely not generalizable, and studies examining how the COVID-19 pandemic impacted the lives and livelihoods of sex workers in India remain extremely limited [14, 31, 32].

### Gendered Stress Proliferation Model

**Fig. 1:**
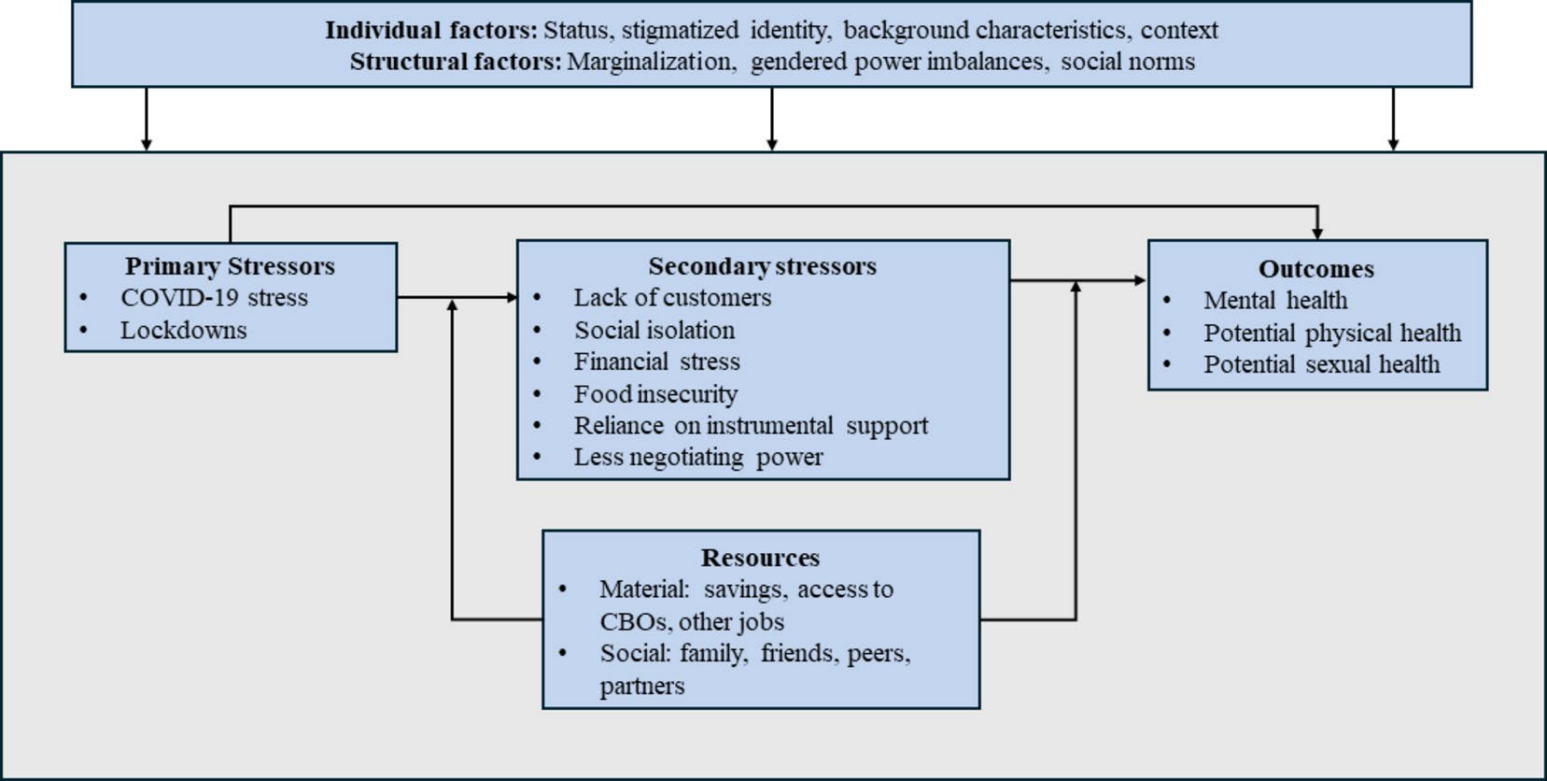
Gendered stress proliferation model for sex workers in India during COVID-19 pandemic and lockdowns

Due to the pandemic’s impact on multiple areas of life, we employ Pearlin’s 1997 model of *stress proliferation*, which refers to the tendency of stressors in one domain to proliferate into multiple domains (i.e., occupational, social, financial, etc.) [33]. Pearlin describes the original stressor as the *primary stressor* and provides the example of AIDS caretakers in his 1997 paper. As primary stressors expand (i.e., increasing caretaking responsibilities) or persist over time, *secondary stressors* arise, for example, the need to quit one’s job to be a full-time caretaker and the resulting financial insecurity. Both primary and secondary stressors impact mental health outcomes [33]. Pearlin’s stress process model describes how life events impact mental health [34], and Swendeman, Fehrenbacher et al. adapted this into the gendered stress process model to show how status, access to resources, and stressors impact mental health outcomes within the context of gendered power structures and social norms for people living with HIV in India [35]. Integrating these theories in the context of our results, we propose a Gendered Stress Proliferation Model applied to the experience of sex workers in India during the COVID-19 pandemic and lockdowns. Figure 1 depicts our integrated model in which primary stressors lead to secondary stressors that together impact health outcomes. The paths from primary stressors to secondary stressors and from secondary stressors to health outcomes are moderated by access to resources, and the entire system is influenced by individual-level identity factors and structural factors such as marginalization and gendered power imbalances.

Stress proliferation has been used within the context of systemic marginalization to examine health outcomes, including for mental health [36]. This framework has been used to examine race-related stressors [36], racial/ethnic discrimination and citizenship status [37], and impacts of racism over the life course [38]. It has also been used to examine health outcomes resulting from intersectional marginalization, such as racial and gender discrimination [39] and gender and socioeconomic status [40]. Stress proliferation has also been used to study downstream health impacts of the COVID-19 pandemic. For example, Secinti et al. used stress proliferation to examine stressors stemming from COVID-19 on Syrian refugee adolescents in Turkey and found that secondary effects, including on financial security, employment, and food access, were more strongly related to PTSD than primary effects of the pandemic [41]. Other studies of stress proliferation in the context of COVID-19 have examined the pandemic’s impact on children’s behavioral problems [42], anger among US adults [43], work environment among healthcare workers [44], and discrimination and mental health among people with disabilities [45]. While the existing literature has emphasized quantifying the impacts of stressors and mediation pathways from primary to secondary stressors to health outcomes, qualitative descriptions of the meaning of stressors and how they are experienced by community members are lacking.

### Present Study

Data on the pandemic’s impact is needed to inform new and ongoing interventions to support sex workers and other marginalized groups. Such data is also needed to better understand the downstream effects and personal economic shocks resulting from COVID-19 prevention policies such as strict lockdowns, stay-at-home orders, and transportation system shutdowns. The present study will address this gap using the stress proliferation model and a qualitative approach to examine the impact of the COVID-19 pandemic and lockdowns on the lives and livelihoods of female, male, and transgender sex workers in Kolkata, India.

## Methods

The research team developed a qualitative interview guide with primary questions and additional probing questions focused on: (1) the impact of COVID-19 pandemic and lockdowns on sex workers generally, (2) the impact on the lives of the participants, (3) the impact on the lives of participants’ family members, (4) the impact on the participants’ friends and co-workers, and (5) participants’ perceptions of and experiences with COVID-19 vaccines (see supplementary materials 1). Individual in-depth interviews were conducted with 30 female, 5 male, and 5 transgender sex workers in Kolkata, West Bengal between June and September 2022 (*N* = 40). Participants were recruited from the Sonagachi district, India’s largest red-light district, through Durbar using existing community networks. Inclusion criteria for female, male, and transgender participants was as follows: self-reported age 18 or older, and self-reported ever engaging in sex work. All participants provided voluntary informed verbal consent for study participation. Verbal consent was used because of low literacy rates in this community and greater protection of confidentiality compared to signed consent (per UCLA IRB guidelines). To obtain verbal consent, interviewers verbally explained the purpose of the research and the interview process then asked whether the interviewee was willing to take part in the interview. For those who consented verbally, the team documented that verbal consent was provided and proceeded with the interview. Durbar research staff conducted in-person interviews with community members. Interviews were conducted in Bangla in a private room in the drop-in centers in Sonagachi and in the nearby Durbar office. Interviewers took detailed notes on participant responses for each question. Notes were translated to English for analysis. Each interview lasted approximately 25–30 min. Data was de-identified to ensure participant confidentiality. The de-identified and translated data was uploaded into Dedoose, a qualitative and mixed-methods analysis software.

The project coordinator reviewed the first 5 transcripts and created a preliminary code tree using an inductive approach to cover all themes present in the data. This preliminary codebook was used to create a Dedoose training module to train members of the analysis team on code application to establish inter-rater reliability and consistency between team members in code application. All analysis team members completed training on study context and qualitative analysis and passed the training module with a pooled kappa score of 0.80 or above before coding new data [46]. The average kappa score across all coders was 0.88. Transcripts were blindly coded by two team members each. The analysis team met weekly to discuss code application strategy and make updates to the code tree as needed, including to modify code definitions, add new codes, and create child codes. Due to the variety of themes identified in the transcripts, the codes were split up into separate manuscript topics. This manuscript examines the impact of the COVID-19 pandemic and lockdowns on the lives and livelihoods of sex workers in West Bengal, India. All themes relevant to this research question from the original code tree were included in this paper. Following completion of coding, codes were divided amongst the members of the analysis team for review of representative excerpts. For their assigned codes, each team member reviewed all excerpts coded as that code and wrote a qualitative summary of how the theme appeared in the data, how it was discussed by participants, and how it answered the research question. During this review, team members also flagged particularly insightful quotes as illustrative quotes. Qualitative summaries of each code along with illustrative quotes are presented below following the proposed Gendered Stress Proliferation Model.

## Results

40 participants were recruited by Durbar staff. 30 participants identified as cisgender female sex workers, all of whom worked in brothels in the Sonagachi district of Kolkata. 5 participants identified as cisgender male sex workers, and 5 identified as transgender female sex workers. Interviews were conducted in two drop-in centers in Sonagachi.

Participants described cascading effects of the COVID-19 pandemic and lockdowns on their lives and livelihoods. Nine interrelated themes emerged from the data. Of these, six were initially identified when the codebook was first developed: (1) First versus second lockdown, (2) Lack of customers, (3) Financial stress/lack of income, (4) Food shortages, (5) Receiving support, (6) Mental health. As coded excerpts were reviewed and analyzed, the code definitions were modified to best represent the data and new codes were added to capture distinct concepts. This resulted in the re-conceptualization of the ‘first versus second lockdown’ code to ‘COVID-19 pandemic and lockdowns’ and the addition of 3 emergent codes: (1) ‘Social isolation’ emerged from excerpts originally coded as ‘first versus second lockdown’ and ‘mental health’, (2) ‘Decreased negotiating power’ emerged from excerpts previously coded as ‘lack of customers’ and ‘financial stress/lack of income’, and (3) ‘Providing support’ emerged from excerpts originally included in the ‘receiving support’ code. Thus, the final code tree included 9 codes: (1) COVID-19 pandemic and lockdowns, (2) Social isolation, (3) Lack of customers, (4) Financial stress, (5) Decreased negotiating power, (6) Food insecurity, (7) Receiving support, (8) Providing support, and (9) Mental health. The final code matrix with all 9 codes is presented in Table 1 below. These codes are described in detail with illustrative quotes below and are organized into primary stressors, secondary stressors, resources, and mental health outcomes per the proposed model.

**Table 1:** Coding matrix

Theoretical Construct	Code	Code definition
Primary stressors	COVID-19 pandemic and lockdowns	Participant describes their experience during the COVID-19 pandemic and/or lockdown periods. This includes comparisons between the first and second lockdown periods
Secondary stressors	Social isolation	Participant describes being unable to see or visit other people (family, children, friends, peers, or other). This includes descriptions of travel restrictions and how this isolation impacted the participant and/or their loved ones
	Lack of customers	Participant discusses having very few or no sex work clients. This may be accompanied by discussions of lack of work and economic struggles. This includes discussions of why this decrease occurred, trends in number of clients over time, and the impact that this had on the participant and/or their peers
	Financial stress	Participant discusses not having enough money. This may be in relation to a lack of customers, but it may be discussed relating to other factors as well (for example, employment outside of sex work or unexpected medical costs). This includes descriptions of financial stability changing before versus during the lockdown periods
	Decreased negotiating power	Participant discusses how the pandemic or related financial difficulties influenced their ability to negotiate with potential sex work clients. This includes negotiation for price, condom use, and other factors related to in-person exchange sex. This also includes how the pandemic/lockdowns influence client behaviors related to price or condom negotiation
	Food insecurity	Participant discusses not having an adequate supply of food for themselves and/or for dependents. This may include discussions of economic hardship, relying on others for food, or food rationing
Access to resources	Receiving support	Participant discusses receiving support from any source during the COVID-19 pandemic/lockdowns. This may include family, friends, non-governmental organizations, political parties, or another source. This includes any type of support including for food, money, or other resources
	Providing support	Participant discusses providing instrumental support or resources to others during the COVID-19 pandemic/lockdowns. This includes children, dependents, family members, partners, friends, peers, or other individuals
Health outcomes	Mental health	Participant discusses their mental health status or describes how their mental health was impacted by the COVID-19 pandemic/lockdowns. This many include descriptions of feeling depressed, anxious, extremely stressed, or struggling with other mental health challenges. This does not include ordinary, daily stressors

### Primary Stressors

#### COVID-19 Pandemic and Lockdowns

India’s first lockdown period in the spring of 2020 was one of the largest and strictest lockdowns implemented anywhere in the world and included transportation system shutdowns and restrictions on all non-essential travel outside the home [12–14, 16]. The second lockdown period in the spring of 2021 was implemented and enforced at the state rather than national level and was less strict despite the steep upsurge in cases and deaths during the pandemic’s Delta wave [13, 17]. Participants described the pandemic and subsequent lockdown periods as devastating and explained that their situation became increasingly dire as the lockdowns and pandemic progressed. They also expressed shock that the lockdown lasted for as long as it did. This is described by Participant 14 below.

*“I had no idea what a pandemic could be like. First one night I heard on TV news that lockdown will start from tomorrow*,* no trains will run for 15 days. Not just trains*,* there was no transportation at all. No one could go out of the house. The Lockdown lasted for almost 4 months instead of 15 days.” (FSW*,* age 32–41)*.

The sudden start of the lockdown coupled with transportation and communication system shutdowns resulted in some participants being unable to see their children, acquire necessary medications, or return home if they were visiting family members when the lockdown was announced. Below, Participant 21 describes the challenges of transportation and financial security stemming from lockdown restrictions.

*Lockdown had a huge impact on my life. Firstly*,* none of us could have imagined that the lockdown would last for so long. The lockdown was announced for the first time by the government for just fifteen days*,* and the days of lockdown kept increasing. After three months of continuous lockdown situation*,* when the lockdown was lifted*,* another trouble started again*,* because then local trains were not allowed*,* so it cost a lot of money to come to brothel or back home. I spent the saddest days of my life sitting at home during the lockdown. (FSW*,* age 42–51)*

During the first lockdown period, no customers were allowed to travel to brothels, and participants described increasing financial distress, food insecurity, and disruptions to children’s education. After a brief normalization period over nine months, the second lockdown was announced, but most participants expressed that the impacts of the second lockdown were not as severe as the first because they were more prepared and restrictions less strict so they were able to see some clients. Below, Participant 11 describes how her experiences during the first lockdown motivated her to take precautions for the second.

*I got into a lot of trouble because the first lockdown started suddenly. A few days before the first lockdown*,* I bought a [plot of] land with as much money as I had*,* so I didn’t have any money at the time of the lockdown. So at that time*,* I was unable to pay the tuition fees for the girls’ education*,* just as there was a problem in getting food. When the first lockdown was over*,* I came back to the brothel and joined the trade again. During the second lockdown*,* the difficulty was not so much because I understood in advance that there might be a lockdown*,* so I saved some of the money which I earned. (FSW*,* age 32–41)*

### Secondary Stressors

#### Social Isolation

Due to stay-at-home orders, as well as transportation and communication system shutdowns, participants experienced intense periods of isolation from family, friends, and peers. Participants were often stuck at home alone, which, in some cases, generated significant mental distress. Male and transgender participants also discussed arguing with family members and unhealthy substance use as negative results of social isolation. Below, Participant 34 describes excessive alcohol consumption resulting from the loneliness of isolation.

*“I was very upset mentally. I sat at home very lonely and felt so bad that I couldn’t sleep at night. From morning till night*,* I used to spend my time drinking alcohol. I could not sleep in the morning*,* noon*,* afternoon*,* even at night without drinking alcohol. I used to be drunk all day and night. I felt that being alone in that state often made me feel mentally ill. But even after the lockdown*,* it took me a long time to reduce my alcohol consumption. Now I don’t drink as much as I used to. But it took time and trouble to control myself. Even during the lockdown*,* I could not get food*,* but I used to get money for alcohol by borrowing money from anywhere.” (Transgender SW*,* age 22–31)*.

Additionally, several participants described being unable to see their children during the lockdown. In some cases, the shutdown of transportation systems coupled with travel restrictions prevented sex workers from visiting their home villages for extended periods of time. Below, Participant 28 describes being separated from her daughter during the lockdown.

*“My younger daughter used to call me every day asking me to come back home but I could not return home as the communication system [for transport] was totally off. After about three months*,* I somehow contacted the police and hired a car and went back to the village. I couldn’t sleep at night for the first three months of the lock down in the Brothel*,* watching the news of people’s deaths on TV all day and thinking that if I get infected with Corona*,* I won’t be able to see my daughter’s face again.” (FSW age 32–41)*.

#### Lack of Customers

All participants emphasized that the COVID-19 pandemic and lockdowns resulted in an inability to work due to very few or no customers. The lack of customers and inability to work was often discussed within the context of significant financial struggle. Participants also described strong distinctions in their experiences between the first and second lockdown periods. All participants recalled having few to no customers during the first lockdown but having more customers during the second. For example, Participant 2 described differences between the first and second lockdown and highlighted the relationship between inability to work, financial stress, and food insecurity.

*“In the first lockdown all the sex workers had a hard time*,* for several days I was just eating parched rice. No customer could come here so there was no income. I survived on help at this period. My condition was a bit better from the first lockdown to the second lockdown. In the second phase*,* [a] few customers came into the red-light area*,* so I was able to earn some income.” (FSW*,* age 42–51)*.

A few participants identified reasons why customers did not come to brothels during the lockdowns. These reasons included restrictions on movement enforced by the government and police, financial stress experienced by potential clients, and clients’ fear that they would contract COVID-19. Below, Participant 4 emphasizes that even in the second lockdown these factors continued to result in fewer customers.

*“I couldn’t make any income because I was at home in the first lockdown*,* but I was able to make some nominal income because I was in the sex trade in the second lockdown. In the second lockdown*,* there was very little business in the sex trade because customers rarely came here for fear of contracting Corona. In the Corona situation*,* the customer’s income also decreased.” (FSW*,* age 42–51)*.

#### Financial Stress

As a result of the lack of customers, participants described significant financial hardship during and after the lockdowns. Many participants described differences in their income and financial status between the first and second lockdown. Most discussed having less income during the first lockdown compared to the second due to stricter restrictions during the first lockdown period. Financial hardship was further exacerbated for those who experienced additional financial burdens on top of the COVID-19 pandemic due to preexisting economic vulnerability. For example, below, Participant 22 emphasizes the financial insecurity stemming from the lockdown that was exacerbated by medical costs leading up to the lockdown.

*“A few days before the first lockdown*,* I gave birth to a daughter. I had already abstained from sex act for several months for the delivery. Moreover*,* I had spent a lot of money during the birth of my daughter*,* so I did not have any money during the lockdown period. My husband used to work in a biscuit factory. Since it was an emergency job*,* the factory was running even during the lockdown. At that time*,* I had no income*,* I had to support my family from my husband’s income.” (FSW age 42–51)*.

Some participants, particularly the male and transgender sex workers, reported having additional sources of income in addition to sex work. In some cases, participants reported that they were still able to work their primary job during the lockdown which allowed them to be more financially stable when they were not able to continue sex work. Others, such as Participant 38, described losing their primary job as well as their work as a sex worker.

*“I used to work in the sales department in a company. After the lockdown started*,* that company was closed. Not only that*,* I didn’t get my salary for that said month. After that I spent my days in great financial trouble because at that time*,* I could not earn any money by providing sexual services to any customers. All the ways of income were closed.” (Male SW*,* age 22–31)*.

In response to significant financial distress, participants discussed their reliance on prior savings (if they had any), support from customers or family members, and food rations. Participants often stated that without such support, their income or savings would not be sufficient for them and their dependents to survive the entirety of lockdown. This distress and the importance of external support are highlighted by Participant 25.

*“…my husband died during the first lockdown. On one hand*,* the pain of not having any income for the whole day during the first lockdown*,* on the other hand*,* the pain of losing my husband*,* I almost broke down mentally. After losing my husband*,* the desire to work seemed to have disappeared. After that*,* even though the lockdown ended gradually*,* the customers hardly came at all. There were maybe two or three customers throughout the whole day. Some days there was not a single customer. As a result*,* the income was less*,* but somehow*,* I managed to support my daughter. In those days*,* the reliefs given by various clubs or political parties or Durbar helped us a lot. After the first lockdown*,* there were very few customers for about 3 to 4 months*,* then when the situation gradually started to normalize*,* the second lockdown started again.” (FSW age 42–51)*.

#### Decreased Negotiating Power

Some participants discussed challenges negotiating price or condom use with clients because of the COVID-19 pandemic, lockdowns, and related vulnerabilities. A few participants, including Participants 9 and 30 below, described situations in which customers paid less than they normally would. Price negotiation was exacerbated by the lack of customers during this time.

*“Some days I sat all day but did not get a single customer. Taking advantage of the situation*,* the customers used to pay less money.” (FSW age 42–51)*.

*“Although some customers would come here*,* they would take advantage of the situation and pay less. Some*,* who previously paid Rs. 500 [6.06 USD]*,* at that time paid Rs. 200 [2.24 USD]. I even had to have sex on receiving Rs. 30 [0.36 USD] only.” (FSW age 42–51)*.

The pandemic’s impact on condom negotiation was described by a few participants. Although a large majority of participants, including Participant 30 below, described very consistent condom use, Participant 13 described having sex without a condom to earn more money because of the financial stress of the lockdown.

*“I have never had a sexually transmitted disease. I have never had sex without a condom. I knew*,* in that situation*,* in case of venereal disease*,* there would be no proper treatment anywhere*,* so I was worried about myself.” (FSW*,* age 42–51)*.

*“There were almost no customers in those tough situations*,* so sometimes I was forced to have sex without a condom in the hope of getting some extra money. So*,* when I got sexually transmitted disease once*,* I went to the government hospital and got medicine. After that*,* I used to take those medicine from Durbar clinic and I am now healthy after consulting the doctor regularly.” (FSW*,* age 52–61)*.

#### Food Insecurity

Participants described intense food insecurity during the lockdowns which stemmed directly from an inability to work and lack of income. Many participants described this situation as life-threatening and emphasized a real fear that they or their family members could die of starvation. This caused significant distress among participants, with many reporting a growing inability to care for their families and feed their children.

*“We did not have much saved money*,* as we live on daily income…The first one month of the first lockdown was not too bad because all the money we had was spent on eating and drinking. But after a month when the time of the lockdown started to increase*,* then we started to feel like what to do now? Now there is no money*,* how will I run the family*,* what will I feed my son?…By God’s infinite grace*,* I could manage to get cooked food from some club people who were distributing cooked food on the street and ate it. I also managed some dry food and cooked food from Durbar.” (FSW age 42–51)*.

As described in the narrative above, some participants had savings that protected them for a limited time from inability to work and food shortages. Other participants did not have any savings, so the lockdown and resulting inability to work immediately affected their access to food for themselves and their dependents. Some participants, such as Participant 15 below, also described the exacerbation of food insecurity by travel restrictions, but also emphasized the necessity of prioritizing food access.

*“[In the village] I managed my life somehow by getting government ration and panchayat relief. A few days later a friend from brothel called me and told me to come here by staff special train. Since then*,* I used to travel by the staff special train every day*,* but I was very afraid if the police would catch and punish me. In this way*,* day by day*,* the fear slowly disappeared. At that time*,* it seemed that it was more important to go outside the house in search of food than to stay at home.” (FSW age 42–51)*.

### Access To Resources: Receiving and Providing Instrumental Support

#### Receiving Support

In response to dire financial situations, participants often discussed relying on external sources of support, including family, friends, non-governmental organizations (including Durbar), local political party clubs, wealthy individuals, and the government. Reliance on external support was especially discussed in the context of the first lockdown, when there were no customers, and most participants did not have a source of income. Several forms of instrumental support were discussed by participants, but most emphasized the importance of food rations, in particular. Many participants, such as Participant 14 below, stated that, without such support, they and/or their family members would have starved.

*“Basically*,* I used to depend on relief food. In that pandemic situation*,* I was dependent on the government ration apart from the relief given by various clubs*,* panchayet also. In the second lockdown*,* the sex trade was not completely closed. So*,* I was able to make some income at that time.” (FSW*,* age 32–41)*.

In response to severe food insecurity, many participants discussed their growing dependence on outside sources for food distribution including community-based organizations and rations from the government, friends, political clubs, or wealthy individuals. This external support was often described as the only way participants could obtain food during the lockdown periods, and many described this support as lifesaving.

*“The factory where my son worked was also closed due to the lockdown*,* so it became very difficult for me to get food for both of us. During the lockdown*,* various clubs*,* political parties*,* apart from Durbar*,* provided dry food and cooked food many times. The relief received from different places saved my family during pandemic situation.” (FSW*,* age 52–61)*.

Besides food support, some participants described receiving financial support, which often came from family, friends, fixed/established partners and/or regular customers. Many participants discussed having to support their family, being the sole income earner in their family, or having to send food and money back to their family in their home village. As a result, outside monetary support from sources beyond family was extremely important to help participants with these responsibilities when they could not work. For example, Participant 26 discusses reliance on financial support from regular customers when she was not able to work.

*“I got through the first three to four months of the lockdown with whatever savings I had but then deep problems started. Then I was forced to borrow money from two very familiar customers of mine to run the family. Also*,* a familiar customer helped me many times with dry food. If we don’t earn every day*,* the family can’t survive*,* there is no income for 6 or 7 months continuously. Towards the end of the lockdown*,* I felt very helpless as I had to ask others for money to run my family.” (FSW*,* age 32–41)*.

Although many participants described receiving some forms of instrumental support, others stated that they did not receive any help during this time. Below, Participant 12 describes not receiving assistance and attributes this to extreme and widespread financial insecurity.

*“In that difficult situation*,* none of the people I asked for help helped me. Actually no one had the ability to help anyone during the lockdown. Everyone’s economic situation was bad.” (FSW*,* age 42–51)*.

### Providing Support

While most participants described receiving instrumental support from an outside source, some also described providing support for others, especially dependents, family members, or sexual/romantic partners who could not support themselves. Below, Participant 33 describes providing food support to his established sexual/romantic partner.

*“No one other than my fixed partner asked for any help. I gave him all the dry food I got from various clubs and NGOs.” (Male SW*,* age 22–31)*.

Due to the widespread financial difficulties during the pandemic and lockdowns, many participants stated that they were not able to provide support for others during this time or stated that nobody asked them for support because their friends and family were aware that they had nothing to spare. Below, Participant 30 describes an inability to provide support for others.

*“Nobody asked me for help at that bad time because everyone knew I didn’t have the financial capability to support anyone.” (FSW*,* age 42–51)*.

While many participants were not able to provide support to others, some described situations in which they provided multiple forms of support to care for close family. Below, Participant 29 describes multiple forms of instrumental support to take care of her family and son.

*“I have only one son who is mentally ill…He is now 17 years old. But his mental condition is almost like a 3–4 years old child…So*,* the difficult situation during the first lockdown cannot be overstated. 90% of my monthly income goes to buy medicine for my son and with the remaining money I used to pay for food and house rent. I didn’t have much savings. So*,* I was able to procure medicine in the first month…But from the next month there was no money to buy medicine. So*,* I went to my father’s house leaving the house completely. I rented a car and went to the village. I have a room there and lived there and supported my family with what I got from the government ration of rice and wheat. My brothers gave some money to buy medicine for my son. I also did various sewing jobs there to support the family and son’s medicine expenses.” (FSW*,* age 32–41)*.

### Health Outcomes

#### Mental Health

Participants discussed mental health challenges including feelings of extreme stress, depression, and anxiety stemming from the COVID-19 pandemic and lockdowns. These mental health challenges extended beyond typical daily stressors and were described as resulting from fear of contracting COVID-19, lockdown restrictions, income instability, social isolation, and other areas of concern throughout the lockdown. Below, Participant 30 discusses the initial relief resulting from a few days off work, followed by intense mental distress as the lockdown progressed.

*“At first*,* I was very happy for a few days during the lockdown*,* but as the days went on*,* my mood started to deteriorate*,* because there was no income at that time. I didn’t like sitting at home all day without any income*,* I became irritable without going out. Then I started thinking about how to support my family*,* pay the rent*,* because by then all my savings were gone. I couldn’t sleep at night worrying about the end*,* sitting in a chair for many nights and looking out of the window*,* wondering when everything would be the same again. During this lockdown my son and daughter were at home in the village. None of them could come here. And I couldn’t go home either*,* even if I tried. [I] spent almost 6 months in lockdown here alone at home.” (FSW*,* age 42–51)*.

As highlighted in the narrative above, participants discussed mental health largely in the context of lack of income, with financial suffering during the lockdown triggering intense worry about providing for other family members, including their children. Some participants stated that their feelings of distress and/or anxiety were exacerbated by the constant overwhelming news of illness and death. Below, Participant 22 describes the stress of watching the news, and Participant 31 describes how his other job working in healthcare contributed to his mental distress.

*“I became very worried because of staying at home for a long time during the lockdown and not having any income. Along with that*,* there was the fear of getting infected with Corona*,* what would happen if I or my children became positive. At that time*,* I did not want to watch TV because when I turned on the TV*,* it would show the news of people dying all around. The atmosphere was suffocating to me. Those difficult days seemed to be unending.” (FSW*,* age 42–51)*.

*“I also work as a health worker in a private hospital. I had to deal with patients having COVID. At that time my mental condition was not good either. Seeing so many patients die in front of my eyes every day*,* I could not sleep all night. Corona instilled a huge amount of fear in everyone’s mind. I was very worried about my family.” (Male SW*,* age 22–31)*.

## Discussion

These results demonstrate the severe, often immediate, impact of the COVID-19 pandemic on the lives and livelihoods of sex workers in West Bengal, exacerbating underlying economic and social vulnerabilities. Participants explained that the strictly enforced shutdown resulted in social isolation, a lack of customers, and an inability to work, especially during the first lockdown period. The inability to work led to a lack of income and significant financial distress, exacerbated by preexisting economic vulnerabilities. Financial instability led to severe food insecurity, inability to afford basic needs, and decreased ability to negotiate price and condom use with clients. Having access to resources, including savings; other jobs; or support from Durbar, other community-based organizations, local political parties, regular customers, and established partners helped buffer the impact of these stressors for some.

Pearlin describes stress proliferation as resulting in an intensifying “constellation of stressors” (pg. 227) [33]. Our results are in line with the stress proliferation model as participants described the cascading impacts of the COVID-19 lockdowns across multiple domains of life. We propose the Gendered Stress Proliferation Model integrating stress proliferation and the gendered stress process model. In this case, the primary stressor is the COVID-19 pandemic and lockdown restrictions. Secondary stressors arose and intensified as the lockdowns persisted. Stress from the lockdowns themselves as well as from secondary stressors negatively impacted mental health outcomes, plus potential impacts for physical and sexual health via food insecurity and decreased ability to negotiate condom use with clients. Access to material and social resources buffered the proliferation of primary stressors into secondary stressors and the pathway from secondary stressors to health outcomes. All relationships in the model are influenced by individual level factors such as intersectional identity characteristics and structural level marginalization (i.e., LGBTQ + identity, gender, caste or class, sex worker status). Our results are also in line with Pearlin’s description of erosion of resources within the context of stress proliferation [34, 47, 48]. As stressors persist over time, the resources of individuals and communities are depleted, which impairs the ability to cope with stressors. In our results, some participants needed to provide support for others but the depletion of resources such as savings over time made this increasingly difficult.

Although participants emphasized mental health outcomes specifically, impacts on physical and sexual health are also implied in our results. Limited food access and reliance on dry food distributed by organizations impacts nutrition and physical health. Additionally, although condom use has been shown to be highly normative in Sonagachi [8, 10], financial vulnerability among sex workers has been shown to reduce negotiating power, including condom negotiation [7, 49]. This is seen in the excerpts presented here in which FSW described accepting clients for lower rates or stated that they had sex without a condom to earn more money. This decreased negotiating power impacts sexual health, including risk for HIV and other STIs. These results highlight the downstream implications of policies intended to prevent the spread of COVID-19. While policies such as travel restrictions and shutting down brothels may have temporarily reduced transmission of COVID-19, the resulting personal economic shocks, particularly for communities that experience marginalization, are severe and may have lasting effects [12, 15, 50, 51].

These results are in line with the limited existing literature examining the impact of the COVID-19 pandemic on sex workers in India, although much of this work has been done outside of West Bengal. Much of the limited literature on this topic has focused primarily on access to HIV-related care and has highlighted the importance of community-led efforts and access to Antiretrovirals (ARVs), including the ability to receive multiple months of ARVs at once for those living with HIV [5, 13, 14, 31, 52]. Among female sex workers, men who have sex with men, and transgender women in Telangana and Maharashtra, India, Pollard et al. found that the pandemic and lockdowns resulted in an inability to engage in sex work due to a lack of customers and an inability to travel due to curfews and a lack of public transportation [14]. The same study documented stress from reduced income; an inability to afford basic necessities; and a need for support services to address financial insecurity, employment, food access, COVID-19 testing and vaccination, mental health, and HIV-related services [14]. Similarly, Reza-Paul et al. documented the same themes of financial stress, food insecurity, and mental health concerns while emphasizing the critical role of community-based organizations in supporting community members during this time [31]. Financial insecurity, reliance on loans, and psychological distress have also been reported among hijras, a sub-culture of transgender women in India [53]. The present study expands upon these findings by providing insights into both the first and second lockdown period, within the context of urban West Bengal.

As has been described elsewhere in the literature, these findings demonstrate that India’s COVID-19 pandemic and lockdowns disproportionately impacted populations with pre-existing vulnerabilities, including communities disproportionately impacted by HIV [15, 21, 50, 51, 53–55]. Studies focused on the general Indian population and Indian workers in informal sectors, have documented widespread unemployment, declines in earnings, increased reliance on loans, and reductions in food intake during the lockdowns [50, 51]. However, these findings demonstrate that sex workers were uniquely impacted by the pandemic and lockdowns stemming from structural inequities, pre-existing vulnerabilities, and continued stigma [14, 31, 32]. For example, while Nath found that employment among informal workers in India fell by 65% during the lockdown [51], participants in the present study nearly universally described a complete halt in sex work during the first lockdown period as clients were unable to enter brothels. Finally, our findings of severe mental health implications stemming from the pandemic and lockdowns are supported by Chakrapanni’s findings among men who have sex with men in India that greater internalized stigma and greater stress related to social distancing contributed to increased depressive and anxiety symptoms [52].

The economic vulnerabilities highlighted in this paper demonstrate the need for financial support interventions to buffer against economic insecurity and its health impacts, even during non-crisis times. These results have implications for practice, particularly regarding the importance of programs and policies to support the well-being of sex workers and to initiate growth of savings that protect against personal and larger scale economic shocks or challenges. This is particularly salient given the pre-existing levels of debt in this community [56] and the exploitative nature of predatory lending and debt bondage among sex workers throughout India [57]. These results also highlight the importance of community-based organizations that provide assistance to sex workers and advocate for community members, as many participants described the importance of receiving critically needed support from community organizations during the lockdown period [58, 59].

A strength of this study is in its use of qualitative methods that center the voices of community members. This strategy reinforces this population’s agency by emphasizing their lived experiences and presenting their experiences in their own words. One limitation of this study is that recruitment of participants occurred in Sonagachi, where Durbar is already highly active in health promotion efforts [7, 8, 60]. Therefore, these results may not be transferable to other sex worker communities without access to these resources or the same history of involvement with health promotion and community development programs. Despite this potential advantage, however, participants in the present study still described debilitating impacts of the pandemic and lockdowns. The dependability (stability over time) of our results may be impacted by the particular historical context of India’s COVID-19 pandemic in 2020–2022. However, our integration of stress proliferation theory and our proposed Gendered Stress Process Model may provide novel ways of understanding how sex worker communities are impacted by future population-level crises. We also note that it is a limitation that the analysis team did not include community members, although it did include investigators who work directly with community members. To increase the credibility of our results, all members of the analysis team (graduate level public health students of diverse racial-ethnic, gender, sexual orientation, socio-economic status, and disability status backgrounds) received thorough training on the community context and history as well as detailed qualitative methods training which emphasized the importance of reflexivity in qualitative analysis.

Another limitation of our results is potential interviewer bias, as interviewers were affiliated with Durbar, so participants who discussed Durbar’s work during the lockdowns may have described these activities more favorably than they would otherwise. However, considering the complex and widespread marginalization that sex workers face and the sensitive topics discussed in these interviews, it was important for interviewers to be affiliated with a long-standing highly trusted organization in the community so that they felt comfortable to share their experiences freely. This long-standing collaboration between community members, Durbar, and the research team at UCLA is also an example of prolonged engagement.

These findings may inform quantitative research priorities and hypothesis development, as parallel survey data are presently being analyzed and were used to triangulate qualitative findings. Future studies could examine the impact of the COVID-19 pandemic on sex workers in other regions of the country, particularly those where street-based sex work, as opposed to brothel-based sex work, is more common. Our proposed model, Gendered Stress Proliferation, may be a useful tool to evaluate how sex workers or other communities that are marginalized based on gendered forces experience large-scale crises. For example, future research may examine the impact of climate crises, future pandemics, or large-scale conflict on sex worker populations using this theoretical framework.

## Conclusion

Sex workers face complex systems of marginalization and occupational risks. Within the context of India’s COVID-19 pandemic and lockdowns, these preexisting vulnerabilities resulted in significant, and often immediate, implications for sex workers’ lives and livelihoods. This study describes the cascading effects of inability to work, financial stress, food shortages, social isolation, negative mental health outcomes, decreased negotiating power, and reliance on external support for sex workers as a result of the COVID-19 pandemic and lockdowns in urban West Bengal. Gendered Stress Proliferation may be a valuable tool to understand how marginalized communities are impacted by national and international crises.

## Electronic Supplementary Material

Below is the link to the electronic supplementary material.

Supplementary Material 1

## Data Availability

Per UCLA IRB restrictions we will make de-identified code-specific excerpts available upon reasonable request. Although sex work is not criminalized in India, sex workers remain highly stigmatized. Thus, even de-identified data may contain information that is highly sensitive. Requests for data may be sent to cchpublications@mednet.ucla.edu and will be reviewed by the data management team at the UCLA Center for Community Health.
