# Supplementary material for: The Impact of the COVID-19 Pandemic and Lockdowns on Sex Workers in West Bengal, India
Source: J Community Health. 2025 Mar 10;50(4):668–81. doi: 10.1007/s10900-025-01452-y (PMC12301274; doi:10.1007/s10900-025-01452-y)
Supplement: Supplementary file 1 — Supplementary Material 1 [file 10900_2025_1452_MOESM1_ESM.docx]

**Durbar In-Depth Interview for Sex Worker Experiences of COVID-19**

1. How has the COVID-19 pandemic and lockdowns affected sex workers generally? [INSTRUCTIONS: *Note that this first question is intentionally general to assess what things are most salient to participants*]

1. How has COVID-19 and lockdowns impacted your life, specifically? [INSTRUCTIONS: *Allow participants to talk about the things most salient to them first. Then use the following PROBES for any areas the participant did not mention or that could be elaborated on for more details*]:
   1. Your livelihood and ability to earn money? How you conduct sex work? How you protect yourself from STIs?
   2. Your health? Did you get COVID-19, and if so, what was your experience like?
   3. What kinds of things have you done to prevent getting COVID-19?
   4. Did you have to ask friends or family for help during this time? What kind of help did you ask for? Did they provide assistance and if so, what kind?
   5. What other kinds of assistance have you received? From who or where?
   6. How were these experiences different or the same during the first lockdown period from March to May 2020, the summer and festival season, and the second lockdown period from April 2021 to present?

1. How has COVID-19 impacted your family? PROBES:
   1. Your children? Their schooling? Other family members?
   2. Did your family members who do not live with you ask for help during this time? What kind of help? Were you able to provide assistance? If so, what kind? If not, why not and how did it affect your family and your relationships with them?
   3. Have any of your family members had COVID-19, and if so, what was their experience like?
   4. What other kinds of assistance have they received? From who or where?
   5. What kinds of things have they done to prevent getting COVID-19?
   6. How were these experiences different or the same during the first lockdown period from March to May 2020, the summer and festival season, and the second lockdown period from April 2021 to present?

1. How has COVID-19 impacted your friends and co-workers? PROBES:
   1. Their work? Their children or families?
   2. Have any of your friends or co-workers had COVID-19, and if so, what was their experience like?
   3. Did your friends or co-workers ask you for help during this time? What kind of help? Were you able to provide assistance? If so, what kind? If not, why not and how did it affect your them and your relationships with them?
   4. What other kinds of assistance have they received? From who or where?
   5. What kinds of things have they done to prevent getting COVID-19?

How were these experiences different or the same during the first lockdown period from March to May 2020, the summer and festival season, and the second lockdown period from April 2021 to present?

1. What are your thoughts and experiences with COVID-19 vaccines? PROBES:
   1. Have you been able to get a COVID-19 vaccine, second dose or booster? If yes, tell me about your experience? If no, what are some reasons you haven’t?
   2. What kinds of concerns did you or do you have about COVID-19 vaccines?
   3. Where do you get information about COVID-19 vaccines? Whom do you trust the most for information? Whom do you trust the least?
   4. Do you think vaccinations should be compulsory or not? Why?
